# Prevalence of Adverse Pregnancy Outcomes, by Maternal Diabetes Status at First and Second Deliveries, Massachusetts, 1998–2007

**DOI:** 10.5888/pcd12.150362

**Published:** 2015-12-10

**Authors:** Shin Y. Kim, Milton Kotelchuck, Hoyt G. Wilson, Hafsatou Diop, Carrie K. Shapiro-Mendoza, Lucinda J. England

**Affiliations:** Author Affiliations: Milton Kotelchuck, MassGeneral Hospital for Children and Harvard Medical School, Boston, Massachusetts; Hoyt G. Wilson, DB Consulting Group, Inc, Silver Spring, Maryland; Hafsatou Diop, Bureau of Family Health and Nutrition, Department of Public Health, Boston, Massachusetts; Carrie K. Shapiro-Mendoza, Lucinda J. England, Centers for Disease Control and Prevention, Atlanta, Georgia.

## Abstract

**Introduction:**

Understanding patterns of diabetes prevalence and diabetes-related complications across pregnancies could inform chronic disease prevention efforts. We examined adverse birth outcomes by diabetes status among women with sequential, live singleton deliveries.

**Methods:**

We used data from the 1998–2007 Massachusetts Pregnancy to Early Life Longitudinal Data System, a population-based cohort of deliveries. We restricted the sample to sets of parity 1 and 2 deliveries. We created 8 diabetes categories using gestational diabetes mellitus (GDM) and chronic diabetes mellitus (CDM) status for the 2 deliveries. Adverse outcomes included large for gestational age (LGA), macrosomia, preterm birth, and cesarean delivery. We computed prevalence estimates for each outcome by diabetes status.

**Results:**

We identified 133,633 women with both parity 1 and 2 deliveries. Compared with women who had no diabetes in either pregnancy, women with GDM or CDM during any pregnancy had increased risk for adverse birth outcomes; the prevalence of adverse outcomes was higher in parity 1 deliveries among women with no diabetes in parity 1 and GDM in parity 2 (for LGA [8.5% vs 15.1%], macrosomia [9.7% vs. 14.9%], cesarean delivery [24.7% vs 31.3%], and preterm birth [7.7% vs 12.9%]); and higher in parity 2 deliveries among those with GDM in parity 1 and no diabetes in parity 2 (for LGA [12.3% vs 18.2%], macrosomia [12.3% vs 17.2%], and cesarean delivery [27.0% vs 37.9%]).

**Conclusions:**

Women with GDM during one of 2 sequential pregnancies had elevated risk for adverse outcomes in the unaffected pregnancy, whether the diabetes-affected pregnancy preceded or followed it.

## Introduction

Pregnancy complications not only have immediate implications for the mother and baby, but also can be a marker for future chronic disease risk. Pregnant women with diabetes are at increased risk for several adverse maternal and infant outcomes including cesarean delivery, prolonged labor, maternal birth trauma, macrosomia, preterm birth, congenital anomalies, and fetal hypoxia (1,2). The most common form of diabetes during pregnancy is gestational diabetes mellitus (GDM), which is defined as carbohydrate intolerance with first onset or first recognition during pregnancy, and complicates 6% to 14% of pregnancies in the United States (3). A woman who develops GDM has a recurrence rate ranging from 30% to 84% in the subsequent pregnancy (4,5) and has a sevenfold increased risk of later developing type 2 diabetes (6). Chronic diabetes mellitus (CDM), defined as type 1 or 2 diabetes diagnosed before pregnancy, complicates approximately 1% of US pregnancies (7). One of the most common complications in diabetic pregnancies is macrosomia. Infants with macrosomia are at an increased risk for future metabolic diseases such as obesity, hypertension, and type 2 diabetes, perpetuating a cycle of poor health and chronic disease (8,9).

Elevated risk of adverse pregnancy outcomes occurs at maternal glucose levels below those resulting in a diagnosis of GDM. The Hyperglycemia and Adverse Pregnancy Outcome Study found continuous relationships between increasing maternal glucose levels and risk of cesarean delivery, birthweight higher than the 90th percentile, neonatal hypoglycemia, and fetal hyperinsulinemia (10). In general, glycemic control during pregnancy can significantly reduce the risk of complications (11). The interconception period is an opportunity for prevention that could improve both short- and long-term outcomes in the mother and child. Understanding patterns of diabetes and diabetes-related complications during pregnancy could inform those prevention efforts. However, epidemiologic research in this area is limited. We are aware of only one study that examined the change in maternal diabetic status between pregnancies and associated newborn outcomes. In 20 Utah hospitals, GDM in a previous pregnancy increased the risk of adverse neonatal outcomes in the subsequent pregnancy, even in the absence of GDM and after controlling for body mass index (BMI) (12). This study builds our previous analysis (5) of diabetes incidence and recurrence during pregnancy by further exploring adverse birth outcomes by diabetes status across pregnancies. We examined large-for-gestational-age (LGA) infants, infants with macrosomia, cesarean deliveries, and preterm births by diabetes status across pregnancies in a diverse population.

## Methods

We used a population-based, maternally linked birth file among women delivering sequential live infants from 1998 through 2007 using the Massachusetts Pregnancy to Early Life Longitudinal Data System (PELL). We restricted our analysis to singleton, consecutive, live-birth, parity 1 and 2 deliveries. Briefly, our data ascertainment and linkage methods involved the following steps. First, we ascertained all singleton deliveries in PELL to the same mother (based on mother’s name, date of birth, and Massachusetts Universal Hospital Identification Number [a combination of the hospital medical record number and patient’s encrypted social security number], n = 181,030 births). Second, we further restricted the data set to first and second parity births (n = 141,233 births). Third, to ensure we had sequential births, we excluded questionable birth chains (ie, in which the date of last prior birth in parity 2 did not match the date of parity 1 birth [n = 6,563]). The final data set comprised 134,760 pairs of sequentially linked parity 1 and parity 2 deliveries.

We identified pregnancies affected by GDM and CDM from hospital delivery discharge (HD) records and birth certificate (BC) data. For HD records, we used *International Classification of Diseases, Ninth Revision, Clinical Modification* (ICD-9-CM) codes 648.0 (CDM, excludes GDM), and 250.0 through 250.9 (CDM without complications [250.0] or with complications [250.1–250.9]). For GDM, we used code 648.8. For the BC data, diabetes status was determined from a checked box indicating maternal CDM or GDM; more than one box could be checked. There was no check box option for “no diabetes” (no DM).

Using a hierarchical system, we classified GDM and CDM diagnoses (5). Briefly, a CDM diagnosis from either source (BC data or HD records) resulted in being classified as CDM. For the remaining records, those with a diagnosis of GDM from either source were classified as GDM. Pregnancies with no diabetes diagnosis from either source were classified as no DM. Using this classification yielded a kappa statistic of 0.69 for both parity 1 and 2 pregnancies and a 98.2% and 97.6% concordance for parity 1 and parity 2 pregnancies, respectively (5). From these classifications, we created 8 categories based on combinations of diabetes status during the 2 pregnancies: No DM/No DM, No DM/GDM, No DM/CDM, GDM/No DM, GDM/GDM, GDM/CDM, CDM/No DM or GDM, CDM/CDM. We considered the combination of CDM/no DM or GDM as implausible and do not report on these in the results.

We estimated the prevalence of 4 adverse pregnancy outcomes: LGA, macrosomia, cesarean delivery, and preterm birth. LGA was defined as birthweight in the 90th percentile or greater for gestational age (13). Gestational age was based on clinical estimates on the BC data when available and on last menstrual period when unavailable. Macrosomia was defined as birthweight higher than 4,000 g, and preterm birth was defined as less than 37 weeks completed gestation. Cesarean delivery was based on HD records (ICD-9 669.7) or BC data. Birthweight, sex, and gestational age were based on BC data alone.

We used descriptive statistics to describe population characteristics overall and by categories of diabetes status. We computed both unadjusted and standardized prevalence estimates for each outcome by diabetes status category. To examine whether adjusting for the differences in population characteristics between diabetes status groups made a difference, we standardized the prevalence estimates for each diabetes category to the distribution of age, race/ethnicity, insurance status, and time between deliveries in the entire analysis data set using logistic regression. We restricted our analysis to deliveries with no missing values of covariates in either parity 1 or 2 deliveries (n = 133,633). For analyses of outcome data, we further limited analyses to women with no missing values of that outcome in either birth.

We examined risk for the 4 outcomes in women with diabetes in one or both pregnancies compared with women who had no diabetes in either pregnancy. *P* values for comparisons between parity 1 and parity 2 deliveries were adjusted for multiple comparison using the Sidak inequality (14). Because comparisons between pairs of diabetes status categories included all possible pairwise comparisons, we adjusted the *P* values for those comparisons using the Tukey-Kramer method (15). We considered a *P* value less than .05 as significant. Finally, we examined prevalence of recurrence of adverse outcomes by diabetes category. Statistical analyses were performed using SAS software V.9 (SAS Institute Inc) for Windows. The study protocol was reviewed and approved by the institutional review board of the Massachusetts Department of Health.

## Results

From 1998 through 2007, we identified 133,633 women with both parity 1 and 2 deliveries who had complete diabetes status and covariate data. Of these women, 6.6% had at least one diabetes-affected pregnancy. For parity 1 deliveries, the mean maternal age was 28 years (Table 1). Almost half (48.9%) of the mothers had 4 or more years of college, and about three-quarters were non-Hispanic white and had private insurance. About one in 5 mothers were foreign-born. For parity 2 deliveries, the mean maternal age was 30 years. The mean time between deliveries was 33 months. From parity 1 to parity 2 deliveries, the percentage of births with LGA, macrosomia, and cesarean deliveries increased, while percentage of preterm births decreased.

**Table 1:** Maternal Characteristics and Pregnancy Outcomes of Study Population (N = 133,633), by Parity at Delivery, Massachusetts, 1998–2007

Characteristic/Outcome	Parity 1 Delivery^a^	Parity 2 Delivery^a^
**Maternal Characteristic**		
**Age, y**		
<20	16.4	14.9
20–24	12.1	2.9
25–29	29.2	20.9
30–34	32.5	37.6
35–39	9.2	20.2
≥40	0.7	3.5
Mean age, y	27.6	30.4
**Education (years completed)**		
<High school diploma (<12)	11.8	7.8
High school diploma (12)	19.6	21.2
Some college (<4)	19.7	20.9
College degree or more (≥4)	48.9	50.1
**Race/ethnicity**		
Non-Hispanic white	76.7	77.0
Non-Hispanic black	5.4	5.5
Hispanic	9.8	9.6
Asian	6.2	6.2
Native American	1.9	1.7
**Private payment source**		
Yes	73.5	72.9
No	26.6	27.1
**Foreign-born**	19.7	19.7
**No. of months between deliveries**		
≤12	1.9	
13–24	33.5	
25–36	33.5	
37–48	16.1	
≥49	15.1	
Mean no. of months between deliveries	33.2	
**Pregnancy Outcome**		
**LGA (≥90th percentile; gestational age, 30–44 wks)**	8.9	12.8
**Macrosomia (>4,000 g)**	9.9	12.6
**Cesarean delivery**	25.5	28.0
**Preterm birth (<37 wks)**	8.0	7.1
**Gestational age, weeks**		
22–27	0.4	0.2
28–33	1.5	1.1
34–36	6.2	5.7
37–38	20.7	27.3
39–41	65.3	61.7
42–45	6.1	3.9
Mean gestational age, weeks	39.1	38.9

Abbreviations: LGA, large for gestational age.

a Values are reported as percentages unless otherwise indicated.

### Maternal characteristics and pregnancy outcomes by diabetes status

Maternal characteristics varied by categories of diabetes status across pregnancies (data not shown). Compared with women with no diabetes during either pregnancy, women with CDM or GDM during either pregnancy tended to be older, less educated, other than non-Hispanic white, and foreign-born. Standardizing our results did not change any of the subsequent findings. Therefore, for the rest of the results, we present the unstandardized findings.

We summarize findings about pregnancy outcomes according to diabetes status categories.

**1. No diabetes during either pregnancy.** Compared with women with no diabetes during either pregnancy (no DM/no DM), women with GDM or CDM during any pregnancy had higher prevalence of all adverse pregnancy outcomes (all were significant except LGA parity 1, GDM/no DM; LGA parity 2, no DM/CDM; macrosomia parity 1, GDM/no DM and GDM/CDM; macrosomia parity 2, no DM/GDM and no DM/CDM) (Table 2). Restricting LGA births to term deliveries did not substantially change prevalence estimates (data not shown). Women with CDM or GDM during either pregnancy also had higher percentage of deliveries at less than 37 weeks completed gestation than women with no diabetes during either pregnancy (Table 2).

**Table 2:** Prevalence of Pregnancy Outcomes, by Diabetes Status, in Linked Parity 1 and 2 Delivery Pairs, Massachusetts, 1998–2007^a^

Statistic	Total	1	2	3	4	5	6	7	8
		Diabetes Status in Parity 1 and 2 Deliveries							
		No DM/ No DM	No DM/ GDM	No DM/ CDM	GDM/ No DM	GDM/ GDM	GDM/ CDM	CDM/ No DM or GDM	CDM/ CDM
**LGA (≥90th percentile)**									
n	131,438	122,800	3,202	434	1,892	1,911	204	426	569
Parity 1	8.9	8.5	15.1^b^	13.8^b^	9.8	13.8^b^^,^ d	17.7^b^^,^ d	12.2	23.6^b^
Parity 2	12.8	12.3^c^	16.3^b^	14.5	18.2^b^^,^ c	22.4^b^^,^ c ^,^ d	28.9^b^^,^ d	18.8^b^	38.7^b^^,^ c
**Macrosomia (>4,000 g)**									
n	133,376	124,542	3,292	454	1,923	1,935	207	433	590
Parity 1	9.9	9.7	14.9^b^	14.1^b^	10.5	12.8^b^	14.0	12.0	19.0^b^
Parity 2	12.6	12.3^c^	14.1	12.8	17.2^b^^,^ c	19.4^b^^,^ c	24.6^b^^,^ d	17.1	30.2^b^^,^ c
**Cesarean delivery**									
n	133,435	124,596	3,294	454	1,922	1,938	207	434	590
Parity 1	25.4	24.7	31.3^b^	38.3^b^	35.7^b^	39.2^b^	42.0^b^	34.1^b^	56.8^b^
Parity 2	28.0	27.0^c^	38.3^b^	44.7^b^	37.9^b^	45.2^b^^,^ d	55.6^b^^,^ d	40.1^b^	65.4^b^
**Preterm birth (<37 wks)**									
n	133,445	124,604	3,294	454	1,924	1,938	207	434	590
Parity 1	8.0	7.7	12.9^b^	14.5^b^	10.3^b^	10.6^b^	18.4^b^^,^ d	12.0^b^	21.0^b^
Parity 2	7.1	6.9^c^	9.2^b^^,^ c	13.4^b^	7.1^c^	9.1^b^	9.2^c^	11.1^b^	18.0^b^

Abbreviations: CDM, chronic diabetes mellitus; DM, diabetes mellitus; GDM, gestational diabetes mellitus; LGA, large for gestational age.

a Sample sizes (ie, n) are counts of sequential pairs of births. Standardization did not change estimates based on logistic models adjusted for the following covariates: mother’s age, race, insurance status, and time between deliveries; therefore, unstandardized estimates are presented.

b *P* < .05 compared with No DM/No DM (adjusted for multiple comparisons).

c *P* < .05 compared with parity 1 within the same column (adjusted for multiple comparisons).

d *P* < .05 compared with GDM/No DM (adjusted for multiple comparisons).

Compared with women who had no diabetes during either pregnancy (no DM/no DM), women who had no DM during parity 1 but GDM (no DM/GDM) or CDM (no DM/CDM) during parity 2 pregnancies had higher prevalence of adverse outcomes in parity 1 (for LGA [8.5% vs 15.1% or 13.8%], macrosomia [9.7% vs 14.9% or 14.1%], cesarean delivery [24.7% vs 31.3% or 38.3%], preterm birth [7.7% vs 12.9% or 14.5%]) (Table 2). Similarly, compared with women with no diabetes (no DM/no DM), women who had no diabetes during parity 2 pregnancy but GDM during parity 1 pregnancy (GDM/no DM) had higher prevalence of adverse outcomes during parity 2 pregnancy (for LGA [12.3% vs 18.2%], macrosomia [12.3% vs. 17.2%], and cesarean delivery [27.0% vs. 37.9%]).

**2. Outcomes between parity 1 and parity 2 pregnancy.** The prevalence of LGA, macrosomia, and cesarean delivery increased from parity 1 to parity 2 deliveries in most diabetes groups (significant only for GDM/no DM, GDM/GDM, and CDM/CDM for LGA and macrosomia) (Table 2). By contrast, the prevalence of preterm birth was lower in parity 2 than parity 1 deliveries in all diabetes categories, but was significant only for those in the no DM/GDM, GDM/no DM, and GDM/CDM categories.

**3. GDM during parity 1 pregnancy.** Among those with GDM during parity 1 pregnancy, those who had GDM/no DM had the lowest prevalence for each of the outcomes examined, in both parity 1 and parity 2 deliveries (Table 2). Women who had GDM/GDM had the next highest prevalence, and women who had GDM/CDM had the highest prevalence in both parity 1 and parity 2, although not all values were significant. Women who had GDM/CDM had higher prevalence of adverse outcomes than women who had GDM/GDM (significant for all outcomes only in parity 2 [data not shown]).

### Recurrence of adverse outcomes

The overall likelihood of repeat LGA and macrosomia was 40.5% and 37.8%, respectively (Table 3). However, for women who had CDM/CDM, the likelihood of repeat was 70.2% for LGA and 64.3% for macrosomia (both significantly different from No DM/No DM). Repeat cesarean delivery overall was 86.0% and was high across all groups. Among women with a cesarean delivery in parity 1, women who had a combination of GDM or CDM during both pregnancies had the highest prevalence of repeat cesarean delivery (91.8%–95.2%) (all significantly different from No DM/No DM). Women who had no diabetes during either pregnancy had the lowest prevalence of repeat cesarean delivery (85.5%). Among women who had a preterm birth in parity 1, women who had a combination of GDM or CDM during both pregnancies had the highest prevalence of a repeat preterm birth (23.8%–40.3%) (significantly different from No DM/No DM only for CDM/CDM). Women who had no diabetes during either pregnancy had the lowest prevalence of a recurrent preterm delivery (20.4%).

**Table 3:** Prevalence of Repeat Pregnancy Outcomes, by Diabetes Status, Massachusetts, 1998–2007^a^

Statistic	Total	1	2	3	4	5	6	7	8
		Diabetes Status in Parity 1 and 2 Deliveries							
		No DM/ No DM	No DM/ GDM	No DM/ CDM	GDM/ No DM	GDM/ GDM	GDM/ CDM	CDM/ No DM or GDM	CDM/ CDM
**Repeat LGA (≥90th percentile)**									
n	11,664	10,448	484	60	186	264	36	52	134
Prevalence	40.5	39.6	39.1	38.3	47.3	55.7^b^	61.1	57.7	70.2^b^
**Repeat macrosomia (>4,000 g)**									
n	13,257	12,061	491	64	201	247	29	52	112
Prevalence	37.8	37.2	34.4	34.4	45.3	52.6^b^	51.7	50.0	64.3^b^
**Repeat cesarean delivery**									
n	33,949	30,729	1,031	174	686	759	87	148	335
Prevalence	86.0	85.5	90.5^b^	89.1	88.9^b^	91.8^b^	93.1^b^	89.2	95.2^b^
**Repeat preterm birth (<37 wks)**									
n	10,704	9,596	424	66	198	206	38	52	124
Prevalence	20.8	20.4	21.7	31.8	17.7	23.8	29.0	23.1	40.3^b^

Abbreviations: CDM, chronic diabetes mellitus; DM, diabetes mellitus; GDM, gestational diabetes mellitus; LGA, large for gestational age.

a Sample sizes (ie, n) are counts of sequential pairs of births. Standardization did not change estimates based on logistic models adjusted for the following covariates: mother’s age, race, insurance status, and time between deliveries; thus unstandardized estimates presented.

b *P* < .05 compared with No DM/No DM (adjusted for multiple comparisons).

## Discussion

Our findings show that adverse pregnancy outcomes were most prevalent for deliveries in which the mother had diabetes during both pregnancies, especially among those with CDM during both pregnancies. In addition, women with diabetes during either of their pregnancies had higher adverse outcomes in both the unaffected and affected pregnancies than did women without diabetes during either pregnancy. For example, women who developed GDM only during their parity 2 pregnancy had a higher prevalence of adverse outcomes during the parity 1 pregnancy than women with no diabetes during either pregnancy. Furthermore, having any diabetes and any poor outcome during parity 1 pregnancy nearly doubles the likelihood of a repeat poor pregnancy outcome in the next pregnancy, even if diabetes is absent during parity 2 pregnancy. Standardizing for age, race/ethnicity, insurance status, and time between deliveries did not change our findings, suggesting that the differences cannot be explained by changes in these selected maternal characteristics across pregnancies.

Our findings are consistent with those of the Utah study (12) that showed a history of GDM without recurrence may still confer an increased risk of LGA, even after adjustment for prepregnancy BMI. Several possible explanations exist for this increased risk even without a formal GDM diagnosis. Screening tests for GDM are imperfect and can miss women with abnormal glucose tolerance (16). Increased glucose levels not meeting the threshold for a GDM diagnosis are also associated with adverse outcomes (17,18). Therefore, women with GDM during one pregnancy may have higher glucose levels during other pregnancies. Other factors such as prepregnancy BMI and gestational weight gain (not measured in our study) are strongly associated with LGA independent of diabetes. Excess gestational weight gain (including women with normal prepregnancy weight) contributes to more than 30% of LGA births and is a predictor of LGA even in the absence of diabetes (19). Women should be encouraged to maintain a healthy BMI before pregnancy and gain weight appropriately during pregnancy to reduce the odds of having adverse pregnancy outcomes. Women with a BMI of 30 kg/m^2^ or higher should be offered and referred to intensive, multicomponent behavioral interventions (20). Furthermore, to prevent excess gestational weight gain, all women should be counseled on appropriate diet and exercise during pregnancy (21).

The National Institute of Health Consensus Conference on Diagnosing GDM raised concern about increasing cesarean delivery rates among women with GDM (22). Having GDM may influence clinical decision making (eg, women with diabetes are at risk for having large babies) and increase the likelihood of a cesarean delivery (23). Medically indicated cesarean delivery rates are higher for women with GDM than for women without diabetes (24). Our data show that cesarean deliveries increased in those deliveries with diabetes in both pregnancies, which may be due to medically indicated deliveries, but this finding needs further exploration to differentiate between elective and medically indicated deliveries.

We found that the prevalence of preterm birth decreased between parity 1 and 2 deliveries among women who had GDM or CDM during either pregnancy, although repeat preterm birth was highest in women who had diabetes during both pregnancies. This finding is inconsistent with the Utah study that reported an increased risk of preterm birth across all categories of diabetes, although this association disappeared when restricting the sample to parity 1 and 2 deliveries, similar to our study (12). Differences in findings could be due to demographic factors such as having an older and more ethnically diverse population in Massachusetts. In Utah, only 3% of women had GDM, and the population is predominantly non-Hispanic white. Other reasons that may account for these differences include changes in clinical practice and a shift in timing of delivery leading to fewer postdate deliveries and fewer late preterm and early-term deliveries (25). Finally, preeclampsia may also play a role; a primary risk factor for preeclampsia is nulliparity, which increases the risk of preterm birth (26).

Although women with GDM are at increased risk for CDM (6), progression to CDM may be reduced with lifestyle interventions. Several randomized trials have demonstrated that weight loss and increased physical activity reduce the risk for CDM in high-risk women, including those with a history of GDM (27–29). However, all of these influential interventions were delivered later in life and did not focus on the inter-conception period. Because women with GDM during their parity 1 pregnancy have a risk of recurrence during future pregnancies approaching 50%, reducing BMI early in these high-risk women not only reduces risk of future CDM (28–30), but also has the added benefit of reducing diabetes-related adverse outcomes during future pregnancies.

A strength of this study was that we used data from a large, population-based cohort of women to examine adverse pregnancy outcomes by diabetes status across pregnancies. However, our study has limitations. First, designation of sequential parity 1 and 2 deliveries may be subject to misclassification error among delivery pairs. However, such misclassification is likely nondifferential. Second, administrative data may underestimate the true prevalence of GDM and CDM diagnoses. Previous research comparing administrative data with medical records report sensitivities for birth certificate data ranging from 46% to 83% for GDM and 47% to 52% for CDM (31). Sensitivities for hospital discharge data ranged from 71% to 81% for GDM and 78% to 95% for CDM. Specificities for both sources are above 98%. Third, we did not have data on maternal BMI, a predictor of both GDM and CDM; therefore, we were unable to examine its additional effects. Finally, our findings may not be generalizable to populations that differ from the Massachusetts population.

Understanding patterns of DM and DM-related complications across pregnancies could inform chronic disease prevention efforts. Both CDM and GDM are risk factors for adverse pregnancy outcomes. Although infants born to women with CDM during both pregnancies had the highest risk for adverse outcomes, infants of women with any history of diabetes during pregnancy still had elevated risk for adverse outcomes in the unaffected pregnancy, regardless of whether the diabetes-affected pregnancy preceded or followed the unaffected pregnancy. These findings suggest a need to carefully monitor women with a history of GDM during a previous pregnancy, regardless of diabetes status during the current pregnancy. To help prevent future development of diabetes and recurrence of adverse outcomes, women should be encouraged to maintain a healthy BMI before and after pregnancy and to avoid excessive gestational weight gain.
